# Isotemporal Substitution Effects of Daily Time Use on Cardiorespiratory Fitness of Children in the OptiChild Study: A Mediation Analysis with Diet Quality

**DOI:** 10.3390/nu16162788

**Published:** 2024-08-21

**Authors:** Youxin Wang, Pingping Zhang, Mingyue Wang, Qinghai Gong, Canqing Yu, Haijun Wang, Antje Hebestreit, Patrick W. C. Lau, Hui Wang, Li Li

**Affiliations:** 1 Department of Endocrinology and Metabolism, The First Affiliated Hospital of Ningbo University, Ningbo 315000, China; youxin@bjmu.edu.cn; 2 Department of Maternal and Child Health, School of Public Health, Peking University, Beijing 100191, China; 2311110212@stu.pku.edu.cn (M.W.); whjun1@bjmu.edu.cn (H.W.); 3 Ningbo Center for Healthy Lifestyle Research, The First Affiliated Hospital of Ningbo University, Ningbo 315000, China; 15071486635@163.com; 4 Ningbo Municipal Center for Disease Control and Prevention, Ningbo 315010, China; gongqinghai@163.com; 5 Department of Epidemiology & Biostatistics, School of Public Health, Peking University, Xueyuan Road, Haidian District, Beijing 100191, China; yucanqing@pku.edu.cn; 6 Key Laboratory of Epidemiology of Major Diseases (Peking University), Ministry of Education, Beijing 100191, China; 7 Department of Epidemiological Methods and Etiological Research, Leibniz Institute for Prevention Research and Epidemiology—BIPS, 28359 Bremen, Germany; hebestr@leibniz-bips.de; 8 Department of Sport, Physical Education and Health, Hong Kong Baptist University, Hong Kong SAR 999077, China; wclau@hkbu.edu.hk; 9 Laboratory of Exercise Science and Health, BNU-HKBU United International College, Zhuhai 519087, China

**Keywords:** cardiorespiratory fitness, diet quality, physical activity, screen time, children

## Abstract

(1) Background: Although daily time-use is associated with diet quality and cardiorespiratory fitness (CRF) in children, their interdependence remains unexplored. This study first examined the associations between reallocating daily movement time and diet quality and CRF, and second the mediating role of diet quality in the relationship between daily time-use and CRF. (2) Methods: This study included 1131 Chinese children (aged 8 to 10 years; median [interquartile range]: 8.5 [8.3, 8.8]) at baseline (September 2022) and 1268 children at the 9-month follow-up (June 2023) from the OptiChild study. Daily durations of moderate-to-vigorous physical activity (MVPA), sleep, and sedentary behavior (e.g., screen time) were self-reported or proxy-reported by parents. Diet quality was assessed via the Diet Quality Questionnaire (DQQ), which uses a 24 h dietary recall and is categorized according to the Global Dietary Recommendations (GDR) score and Food Group Diversity Score (FGDS). The CRF was measured using VO_2max_ after the 20 m shuttle run test. Longitudinal associations between daily time-use, diet quality, and CRF were calculated using isotemporal substitution models. Mediation analyses were used to determine whether diet quality mediated the associations between daily time-use and CRF. (3) Results: Reallocation of 30 min from screen time to MVPA resulted in significant improvements in the GDR score (β baseline = 0.11, *p* = 0.024; β follow-up = 0.26, *p* < 0.001), FGDS (β baseline = 0.11, *p* = 0.006; β follow-up = 0.19, *p* < 0.001), and CRF (β baseline = 0.40, *p* < 0.001; β follow-up = 0.26, *p* = 0.001). Diet quality partially mediated the associations between MVPA, screen time, and CRF. Substituting 30 min of screen time for MVPA led to diet quality mediating a proportion of the association with CRF (GDR score: 11.4%, FGDS: 6.6%). (4) Conclusions: These findings underscore the importance of optimizing daily time-use of MVPA and screen time and improving diet quality to promote physical fitness in school-aged children.

## 1. Introduction

Children’s health and well-being are intricately linked to their daily movement patterns, which include physical activity, sleep, and screen time or other sedentary behaviors [1]. Adequate moderate-to-vigorous physical activity (MVPA), sufficient sleep duration, and shorter sedentary time, especially lower screen time are essential for a healthy lifestyle and can greatly affect children’s well-being and mental health, by improving physical fitness, and reducing the risk for adiposity and cardiovascular diseases [2,3,4].

In order to provide a deeper understanding of cardiorespiratory fitness (CRF) at a young age, the complex interplay between daily movement behaviors, diet quality, and the CRF must be investigated. Childhood diet quality stands as a pivotal factor in influencing the life-long risk of non-communicable diseases (NCDs) [5,6,7]. On the other hand, CRF has exhibited favorable associations with reduced risk of cardiometabolic diseases, adiposity, and improved mental health and cognitive functions among children and adolescents, thus serving as a fundamental indicator in the assessment of health among youth [8,9,10]. Recent studies have revealed that higher levels of MVPA are positively associated with better diet quality and improved CRF among children, whereas increased sedentary behaviors, particularly prolonged screen time, have been linked to poorer diet quality and decreased CRF levels [11,12,13,14,15,16,17]. Given the finite nature of daily time, any intervention that modifies the duration of one activity inevitably affects the time allocated to others. Therefore, understanding how changes in children’s daily movement behaviors influence their diet quality and CRF is essential for developing effective health promotion guidelines and intervention programs tailored to children’s needs.

The isotemporal substitution paradigm, a statistical approach, has emerged as a valuable tool for addressing the constrained nature of daily time allocation [18]. This approach employs linear models to investigate the theoretical effects of substituting one activity for another. While some studies have demonstrated positive associations between reallocating sedentary behavior into MVPA and CRF [19,20,21], no study has examined the effects of the replacement of screen time on these relationships. Moreover, the substitution relationship of one daily movement behavior to another on diet quality remains unknown. Additionally, previous studies investigated associations between daily MVPA duration or screen time and diet quality and CRF and observed that diet quality was related to CRF [22,23,24,25]. Physical activity, especially MVPA, has been shown to increase awareness of healthy behaviors, including better food choices, as active individuals may be more inclined to consume nutritious foods to support their activity levels [26]. This positive feedback loop can enhance overall diet quality. Reducing screen time, which is often associated with unhealthy snacking and consumption of energy-dense, nutrient-poor foods, can decrease the intake of these foods, thus improving diet quality [27]. Furthermore, diet quality itself is a critical determinant of CRF. It is plausible to suppose that MVPA duration and screen time may be related to CRF not only directly but also indirectly through their association with diet quality. Similarly, substituting screen time with MVPA may have significant implications for CRF through its relationship with diet quality, but these relationships require further elucidation. Therefore, we hypothesized that substituting daily time-use among different activities is associated with diet quality and CRF, and that diet quality mediates the relationship between daily time-use and CRF in school-aged children.

The present study aimed to test these hypotheses by comprehensively examining the associations between daily time-use (daily MVPA duration, sleep duration, and sedentary behaviors, such as screen time), diet quality, and CRF in school-aged children at two time points. Our primary objective was to analyze the associations between the substitution of daily time-use from one movement behavior to another and diet quality and CRF. Additionally, we aimed to investigate whether diet quality mediates the relationships between MVPA, screen time, and CRF, and whether substituting screen time with MVPA influences CRF through shaping diet quality.

## 2. Materials and Methods

### 2.1. Study Design and Participants

The data of this prospective study were collected at baseline and 9 months from the “Optimizing Intervention Effects in Children and Adolescents in Ningbo” program (OptiChild study), a cluster-randomized controlled trial focused on assessing the impact of a comprehensive intervention on weight management among third-grade primary school students (Registration No. at clinicaltrials.gov: NCT05482165). Participants were drawn from six primary schools located in the Haishu, Yinzhou, and Zhenhai districts of Ningbo city. Children aged between 8 and 10 years, who were in third grade, were enrolled in September 2022 and completed the follow-up assessment in June 2023. Approval for the program was obtained from the Ethics Committee of the First Affiliated Hospital of Ningbo University (Approval No. 2021-R168), and written informed consent was obtained from all participating students and their primary guardians.

**Figure 1:**
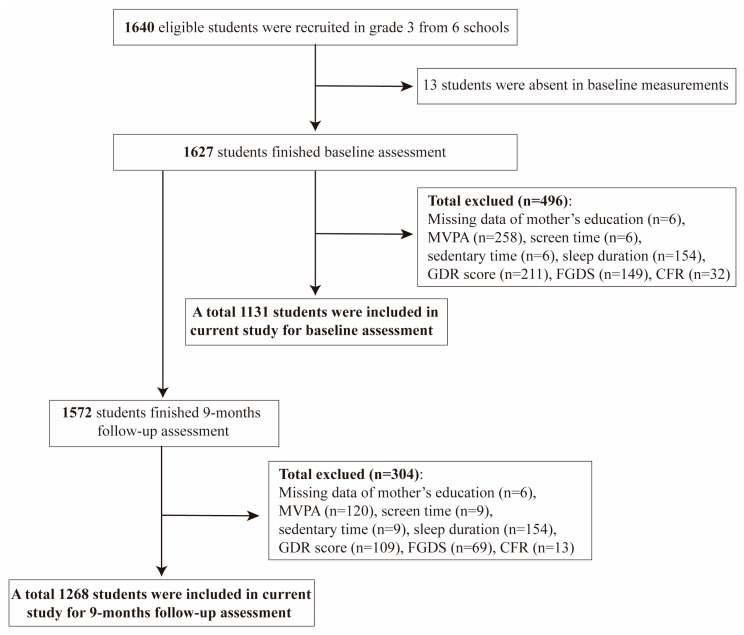
Flowchart for the selection of the participants in current longitudinal study.

The OptiChild study recruited 1640 children, among whom 1627 completed the baseline survey, and 1572 completed the follow-up survey after nine months. After excluding participants with missing data on age, sex, body mass index (BMI), mother’s education, MVPA time, sedentary time, screen time, sleep time, GDR score, FGDS, and CRF, the number of participants with complete baseline data was 1131, while the number of participants with complete follow-up data was 1268 (Figure 1). The mediation analysis was conducted using follow-up data due to higher questionnaire completion rates and quality among children during the follow-up period. The baseline data were used for sensitivity analysis.

### 2.2. Anthropometry and Daily Time Use Measurements

Height and weight measurements were conducted by well-trained health professionals following standardized procedures in September 2022 and June 2023. Height was measured to the nearest 0.1 cm using a mechanical height meter, while weight was measured to the nearest 0.1 kg using a bioimpedance analysis system (InBody770, InBody Co., Ltd., Cerritos, CA, USA), which has been demonstrated in previous studies [28,29]. BMI was calculated by dividing weight (in kilograms) by the square of height (in meters).

The survey process, conducted in September 2022 and June 2023, was supervised by trained research assistants to ensure consistency and accuracy. Parents completed the online questionnaires related to their children’s daily time-use. Specifically, assessments of screen time (hours/day) after school and sedentary time (hours/day) were derived from these questionnaires. In this study, screen time referred to the duration spent by children in front of electronic devices such as watching TV, using mobile phones, computers, tablets, and other electronic devices after school. Sedentary time, excluding screen time, comprised activities such as studying at home after school, attending after-school tutorials, listening to music, playing chess, and reading extracurricular books, among others. 

Children, on the other hand, self-reported their daily MVPA and sleep duration using established and validated questionnaires under the supervision of trained staff. Specifically, using an established questionnaire [30], children self-reported the time they spent sleeping in response to the following questions: “During the past week, what time did you wake up in the morning and what time did you go to bed at night (separately for weekdays and weekends)?”. Sleep duration was calculated between the two time points, falling asleep and waking up the next morning. 

Children’s daily MVPA duration was measured using a self-designed questionnaire adapted from the validated international physical activity questionnaire short form [31], which was applied in our previous DECIDE study [30]. Children self-reported the duration (hours and minutes per day) and frequency (times per weekday or weekend) of all physical activities. The MVPA duration per day was determined by combining moderate leisure-time physical activities (e.g., cycling, fast walking, table tennis, badminton, dancing) and vigorous leisure-time physical activities (e.g., running, basketball, football, swimming, gym fitness exercises). All questionnaires collected data on the daily duration of these activities for both school days (Monday to Friday) and non-school days (Saturday and Sunday). The average daily activity time was calculated using the following formula: [(daily movement minutes on weekdays × 5) + (daily movement minutes on weekends × 2)]/7 = mean movement minutes per day.

### 2.3. Physical Fitness and Diet Quality Measurements

The Diet Quality Questionnaire (DQQ) was completed by the children themselves under the supervision of clinical nutritionists. Diet quality was assessed using the DQQ, where food consumption on the first day of a 24 h dietary recall was categorized into 29 distinct food groups, following the DQQ framework tailored to represent Chinese dietary patterns. This questionnaire effectively captures food group intakes specific to the Chinese population [32,33,34,35]. From the dietary intake data, four indices were derived: (1) the GDR-Healthy score, which includes five global recommendations promoting health-enhancing foods such as fruits and vegetables, beans and other legumes, nuts and seeds, whole grains, and dietary fiber; (2) the GDR-Limit score, which encompasses six global recommendations to restrict certain dietary components including total fat, saturated fat, dietary sodium, free sugars, processed meat, and unprocessed red meat; (3) the overall GDR score, which indicates adherence to global dietary recommendations protective against non-communicable diseases, calculated as the GDR-Limit score—GDR-Healthy score + 9 = GDR score, encompassing all 11 recommendations; and (4) the Food Group Diversity Score (FGDS), which assesses diversity across 10 healthy food groups, including grains, pulses, nuts, seeds, dairy, meat, vegetables, fruits, among others. The scores for the GDR-Healthy and GDR-Limit indices ranged from 0 to 9 points, with the overall GDR score ranging from 0 to 18 points. Similarly, the FGDS ranges from 0 to 10 points. A higher overall GDR score and FGDS indicate superior diet quality.

CRF was evaluated using the 20 m shuttle run test (20mSRT) in September 2022 and June 2023, administered by trained professionals using standardized protocols [36]. This test, extensively employed in the evaluation of CRF among children and adolescents, involved participants running between two markers spaced 20 m apart while synchronizing their pace with audio cues. The test progressed through multiple stages or levels, each lasting approximately one minute, with each stage comprising a certain number of 20 m laps, commonly referred to as shuttles. Initially, the speed was set at 8.5 km/h and gradually increased by 0.5 km/h every minute, with each minute representing a new stage [37]. The test terminated when a child failed to align with the audio signals at the finish line on two consecutive occasions or voluntarily stopped due to exhaustion. The total number of completed laps was recorded, and maximal oxygen consumption (VO_2max_, expressed in mL/kg/min) was estimated using the Léger equation, which is specifically tailored for children aged 6 to 18 years, where a higher VO_2max_ value indicates superior CRF [38].

### 2.4. Statistical Analysis

In this study, we conducted separate analyses on two distinct cross-sectional datasets: one collected at baseline and the other during follow-up. Our primary findings are grounded in the follow-up sample, while the baseline sample serves as a robust validation check for our observations. Descriptive statistics were utilized to characterize the baseline and 9-month follow-up characteristics of the participants. For continuous variables with a normal distribution, mean values and standard deviations (SDs) were reported. For continuous variables with a non-normal distribution, the median and interquartile range (IQR) were used. Counts and percentages were provided for categorical variables. The Shapiro–Wilk test was applied to assess the normality of the data distribution. Multivariate linear regression models were employed to investigate the relationships between daily movement and the GDR score, FGDS, and CFR. Separate models were constructed to assess the association of each daily movement time (sedentary time, sleep duration, screen time, or MVPA time) with the GDR score, FGDS, and CFR. Model 1 explored the association of a single movement time without adjustment for other variables, whereas Model 2 included adjustments for potential confounders such as sex, age, BMI, mother’s education, and schools. For the follow-up data, additional adjustments were made to account for potential confounding effects introduced by different intervention measures within the OptiChild program. Furthermore, in Model 3, all aforementioned movement variables were mutually adjusted to assess their independent contributions. Using the isotemporal substitution approach, we examined the impact of reallocating time from one movement type to another on the GDR score, FGDS, and CFR [18]. For instance, we examined the impact of replacing sleep time with other daily activities. The substitution model is formulated as follows: GDR score/FGDS/CRF = (b1) MVPA + (b2) screen time + (b3) sedentary time + (b4) total daily time + (b5) covariates, where b1–b5 are the coefficients for the respective activities or covariates. By excluding one activity component (e.g., sleep time) from the model, the coefficient (b4) for total daily time reflects the omitted activity component. The remaining coefficients indicate the effect of substituting 30 min of another daily activity for the excluded activity while keeping other activities constant. Regression coefficients derived from these models quantified the differences in the GDR score, FGDS, and CFR associated with engaging in various types of physical activity for a duration of 30 min. In these models, three out of four daily movement variables—sleep duration, MVPA, sedentary time, and screen time—were consistently included, with adjustments made for total time and other relevant covariates [39]. The results are presented as β-values with 95% confidence intervals (CIs) to provide a measure of uncertainty. 

The mediation model was employed to explore whether diet quality mediates the relationships between MVPA, screen time, and CRF, as well as the mediation effects of diet quality on the association between substituting 30 min of screen time for MVPA and CRF. The total effect (TE) of daily movement time and its reallocation on CRF are depicted by a black line annotated with “c” and can be expressed as Equation:

CRF ~ c × daily movement time + covariables. (1)

The direct effect (DE) of daily movement time and its reallocation on CRF are represented by a black line with “c’” annotation. The indirect effect (IE) of daily movement time and its reallocation on CRF are represented by a black line with “a” and “b” annotations. The mediation equation comprises two parts:

GDR score/FGDS ~ a × daily movement time + covariables; (2)

and:

CRF ~ c’ × daily movement time + b × GDR score/FGDS. (3)

The values of a, b, and c represent corresponding regression coefficients, denoted as β values. A significant “indirect role” (mediation) was established when coefficients a, b, and c were significant, with the mediating effects expressed as IE = a × b [40]. The bootstrapping method (1000 samples) was utilized to assess whether the association between daily movement or its reallocation and CRF was mediated by diet quality. The proportion of mediation was calculated as the ratio of IE to TE. All the statistical analyses were performed using R version 4.3.1, and the mediation package in R was employed to estimate the DE, IE, and TE, with statistical significance set at a two-tailed *p* value of <0.05.

## 3. Results

### 3.1. Baseline and 9-Month Follow-Up Characteristics 

Of the 1627 children initially assessed and the 1572 followed up after 9 months, 1131 and 1268, respectively, had complete data for the current analysis (see Figure 1). As presented in Table 1, at baseline, the participants comprised 600 girls (53.1%) and 531 boys (46.9%), with a mean age of 8.5 (8.3, 8.8). The baseline BMI was 15.8 (14.7, 17.7). No significant differences in participant characteristics were noted between the included and excluded participants. The characteristics observed at follow-up remained consistent with those at baseline. 

### 3.2. Independent and Partition Model Analyses

Table 2 summarizes the associations between daily MVPA, sedentary time, screen time, sleep time, CRF, and diet quality, as assessed by GDR scores and FGDS. At baseline, MVPA exhibited a positive correlation with CRF (β = 0.40, 95% CI: 0.23~0.58, *p* < 0.001) and FGDS (β = 0.15, 95% CI: 0.06~0.25, *p* = 0.002), and a marginally significant association with GDR score (β = 0.11, 95% CI: −0.01~0.23, *p* = 0.080) in the unadjusted model (Model 1). These cross-sectional associations were observed at follow-up. Specifically, every 60 min per day spent on MVPA was positively correlated with CRF (β = 0.21, 95% CI: 0.08~0.34, *p* = 0.002), FGDS (β = 0.16, 95% CI: 0.09~0.23, *p* < 0.001), and GDR score (β = 0.15, 95% CI: 0.05~0.23, *p* = 0.002). These associations remained robust even after adjusting for sex, age, mother’s education, BMI, and schools in Model 2, and further incorporating screen time, sedentary time, and sleep time in Model 3. Every 60 min per day spent on screen time showed an inverse association with CRF (β = −0.41, 95% CI: −0.69~−0.12, *p* = 0.005), FGDS (β = −0.33, 95% CI: −0.48~−0.18, *p* < 0.001), and GDR score (β = −0.41, 95% CI: −0.60~−0.22, *p* < 0.001) at follow-up. These inverse associations persisted after multivariate adjustments in Models 2 and 3. Although the results at baseline were not statistically significant, they consistently demonstrated a trend towards inverse associations. Notably, no significant associations were observed between sleep time or sedentary time and CRF, FGDS, or GDR score, except for a significant association between sedentary time and CRF.

### 3.3. Isotemporal Substitution Models

The isotemporal substitution model analyzed the association of reallocating 30 min/day from sedentary time, screen time, sleep time, and MVPA with physical fitness and diet quality, as presented in Table 3. After adjusting for all confounders, replacing 30 min/day of screen time with an equivalent duration of MVPA had a positive relationship with CRF (β = 0.40, 95% CI: 0.26~0.53, *p* < 0.001), FGDS (β = 0.11, 95% CI: 0.03~0.19, *p* = 0.006), and GDR score (β = 0.11, 95% CI: 00.02~0.21, *p* = 0.024) at baseline. These associations persisted at follow-up. Specifically, substituting 30 min/day of screen time with an equal duration of MVPA was positively correlated with CRF (β = 0.26, 95% CI: 0.11~0.42, *p* = 0.001), FGDS (β = 0.19, 95% CI: 0.11~0.28, *p* < 0.001), and GDR score (β = 0.26, 95% CI: 0.15~0.38, *p* < 0.001). Similar results were observed when increasing 30 min/day of MVPA at the expense of sedentary time.

### 3.4. Mediation Analysis

**Figure 2:**
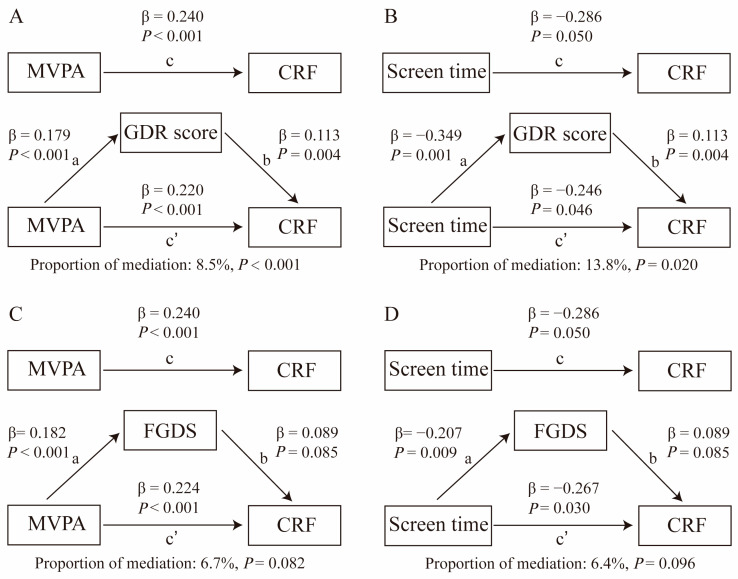
Mediation analysis: contribution of screen time and MVPA on CRF through GDR score and FGDS, adjusting for potential confounders (age, sex, BMI, mother’s education, schools and intervention groups). (**A**) mediation role of GDR score between MVPA and CRF; (**B**) mediation role of GDR score between screen time and CRF; (**C**) mediation role of FGDS between MVPA and CRF; (**D**) mediation role of FGDS between screen time and CRF.

**Figure 3:**
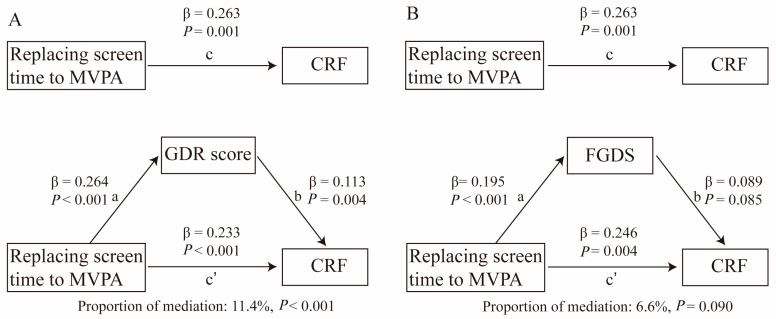
Mediation analysis of GDR score and FGDS on the relationships between 30 min of daily screen time displacing MVPA time and CRF, adjusting for potential confounders (age, sex, BMI, mother’s education, schools and intervention groups). (**A**) mediation analysis of GDR score; (**B**) mediation analysis of FGDS.

The mediation analysis was mainly conducted using follow-up data due to higher questionnaire completion rates and quality among children during the follow-up period. The baseline data were used as sensitivity analysis. Overall, MVPA was positively associated with CRF, while screen time showed a negative association with CRF. Mediation analysis incorporating diet quality (assessed by FGDS and GDR score) revealed that the association between MVPA, screen time, and CRF was mediated through FGDS and GDR score based on follow-up data. Specifically, GDR score mediated 8.5% (*p* < 0.001, Figure 2A) of the association between MVPA and CRF and mediated 13.8% (*p* = 0.020, Figure 2B) of the association between screen time and CRF. FGDS showed marginally significant mediation between MVPA and CRF (*p* = 0.082, Figure 2C), as well as between screen time and CRF (*p* = 0.096, Figure 2D). When reallocating 30 min/day of MVPA with an equal amount of screen time, GDR score mediated 11.4% (*p* < 0.001, Figure 3A) of the association between this reallocation and CRF. Similarly, FGDS showed marginally significant mediation between reallocating 30 min/day of MVPA with an equal amount of screen time and CRF (*p* = 0.090, Figure 3B). These results are consistent with sensitivity analysis, except when analyzing the mediation of diet quality in the association between screen time and CRF, where only a significant trend was observed (Figures S1 and S2).

## 4. Discussion

The present study revealed that increased MVPA duration and decreased screen time were significantly associated with improved GDR score, FGDS, and CRF both at baseline and at the 9-month follow-up. Isotemporal substitution analysis further revealed a positive relationship between substituting screen time with MVPA and these health indicators. Additionally, this study demonstrated that GDR score and FGDS functioned as partial mediators in the relationship between MVPA, screen time, and individual CRF among Chinese children. Notably, when 30 min per day of MVPA was replaced with an equivalent duration of screen time, the GDR score and FGDS continued to serve as partial mediators in the association between this substitution and CRF. To our knowledge, this is the first study that revealed daily time-use reallocation and diet quality and CFR and first to establish the mediating role of diet quality on the relationship between daily movement behavior and CRF.

Accumulating evidence indicates that behaviors play a pivotal role in determining children’s CRF and diet quality. Among these factors, the beneficial impact of MVPA on enhancing CRF is well-documented [41]. Our study findings align with previous research exploring the association between MVPA and this critical fitness component, thereby reinforcing the significance of maintaining adequate levels of MVPA during daily time-use allocation. As advocated by the World Health Organization (WHO), school-aged children should aim for an average of at least 60 min of daily MVPA over a week [42]. Our study also revealed a significant positive association between MVPA and diet quality indicators such as GDR score and FGDS, consistent with prior research [11,12]. This finding suggested that increased physical activity levels are associated with improved dietary habits, possibly reflecting a healthier overall lifestyle. Moreover, our isotemporal substitution analyses conducted at two cross-sectional time points revealed that reallocating 30 min per day of screen time or sedentary time to an equivalent amount of MVPA maintains this positive relationship, reinforcing our findings and highlighting that adjustments in activity patterns can yield meaningful impacts on diet quality. Given the recognized importance of a balanced and diverse diet in mitigating the risk of cardiovascular diseases and dyslipidemia, unhealthy dietary patterns and limited dietary diversity may enhance susceptibility to cardiometabolic diseases [43,44]. Future research should delve into the underlying mechanisms of these associations and explore potential synergies between physical activity and dietary interventions to foster optimal health outcomes for children.

Interestingly, the current prospective study revealed a significant negative association between screen time and GDR score, FGDS, and CRF, whereas other sedentary time excluding screen time exhibited no significant or inconsistent relationships with these variables. Our findings align with current evidence indicating that higher levels of screen time are linked to increased energy intake, poorer diet quality, poorer CRF, and lower quality of life [45]. Screen time may serve as an independent risk factor for GDR score, FGDS, and CRF compared to other forms of sedentary behavior. As screen time is recognized to amplify concurrent snacking behaviors, attributed in part to food marketing, such tendencies might not manifest similarly during other sedentary, non-entertaining behaviors, such as homework, playing an instrument, or drawing, where simultaneous eating is impractical due to occupied hands [46]. Furthermore, reallocating 30 min/day of screen time to an equivalent amount of MVPA resulted in notable improvements in GDR score, FGDS, and CRF observed at two cross-sectional time points. However, reallocating 30 min/day of sedentary time to an equal amount of MVPA did not yield improvements in GDR score and FGDS, except for observed enhancements in CRF during follow-up assessments. Additionally, even after reallocating 30 min/day of screen time to an equal amount of other sedentary time, enhancements in GDR score, FGDS, and CRF were noted. These findings may underscore the superior health benefits associated with replacing screen time compared to other forms of sedentary behavior. Moreover, in school-aged children, sedentary behaviors such as homework and extracurricular activities are often employed to enhance academic performance, posing challenges for parents and teachers to endorse utilizing this time for promoting health through MVPA. In contrast, there is only weak evidence suggesting that small amounts of daily screen use are not harmful [45], making it easier for parents and teachers to support screen time reallocation. Therefore, advocating for reducing screen time to facilitate MVPA and thus enhance overall health may hold even greater practical significance [47].

Another intriguing and novel finding from our study, as uncovered through mediation analysis, is that diet quality appears to partially enhance the association between MVPA and CRF while simultaneously attenuating the association between screen time and CRF in school-aged children. In our findings, we observed that cross-sectionally, the GDR score mediated 8.5% of the association between MVPA and CRF and 13.8% of the association between screen time and CRF. Further evidence from the isotemporal substitution analysis revealed that even after increasing MVPA by 30 min/day at the expense of an equivalent duration of screen time, GDR score continued to serve as a mediator, partially enhancing the relationship between this substitution and CRF. The same mediating role was also observed for FGDS. While increasing physical activity can enhance both diet quality and CRF, the potential mediating role of diet quality in this relationship has not yet been investigated or reported. This knowledge gap hints at the existence of underlying biological mechanisms that could link diet quality, physical activity, screen time, and CRF, warranting further exploration. One plausible mechanism is that high-quality diets rich in fruits, vegetables, whole grains, and plant proteins provide essential nutrients and antioxidants that support optimal metabolic function and cardiovascular health [48,49]. This optimal metabolic state may enhance the physiological responses to physical activity [50], such as carbohydrate metabolism, mitochondrial biogenesis [51], and oxygen utilization [52], thereby potentiating the beneficial effects of MVPA on CRF. Conversely, a poor diet can lead to inflammation, oxidative stress, and impaired vascular function, which may attenuate the positive impact of physical activity on CRF [53,54]. Moreover, diet quality may also modulate the negative effects of sedentary behaviors like prolonged screen time. Sedentary behaviors can disrupt metabolic homeostasis, leading to insulin resistance, dyslipidemia, and endothelial dysfunction [53]. A healthy diet may counteract these negative metabolic changes, reducing their impact on CRF [55]. Additionally, specific dietary components such as antioxidants, omega-3 fatty acids, and dietary fiber can directly influence vascular health and exercise performance [56,57]. These nutrients may further mediate the relationship between MVPA and CRF by promoting vascular health, improving oxygen utilization, and reducing inflammation. Further research is warranted to elucidate the specific biological mechanisms driving these associations and guide targeted interventions to optimize CRF in children. This novel finding emphasizes the importance of considering dietary habits alongside physical activity levels and sedentary behaviors when assessing cardiovascular health outcomes in children. Addressing both physical activity levels and diet quality may offer synergistic benefits for CRF, highlighting the importance of multifaceted health promotion strategies.

Our study has several limitations. Firstly, while the questionnaires employed to assess daily movement behaviors in this study were validated and deemed reliable for children, their precision may not reach the level of accelerometer data. Nonetheless, our study effectively distinguished between screen time and other sedentary time using self-reported questionnaires. Secondly, our survey did not gather information on light physical activity, precluding the observation of the effects of reallocating screen time or other sedentary time to light physical activity on diet quality and CRF. Thirdly, while our study reveals the mediating role of diet quality on the relationship between daily time-use and CRF, the data are cross-sectional, and we cannot infer causality. Future prospective studies are necessary to validate these findings. Finally, in the analysis of the follow-up data, although our study adjusted for the influence of intervention factors, and the interventions did not produce significant changes in CRF, some factors related to the interventions may have influenced the results. Therefore, the findings of our study require further validation in naturally followed populations and through experimental research to establish causality.

Despite the aforementioned limitations, the present study possesses several noteworthy strengths. Firstly, it stands out for employing a novel analytical approach to investigate the theoretical impact of a fixed duration of screen time combined with a fixed duration of MVPA on children’s CRF and diet quality. This approach offers a unique perspective on how different patterns of daily time-use allocation affect crucial health indicators in children. Moreover, the study is pioneering in its examination of the mediating role of diet quality in the relationship between MVPA, screen time or their daily time-use allocation, and CRF. This examination offers valuable insights into the intricate interplay between daily time-use, diet quality, and CRF on health. Furthermore, different from a single cross-sectional study, this research conducted analyses at two cross-sectional time points using baseline and follow-up data, thereby enhancing the reliability and robustness of the findings. The use of longitudinal data allows for the verification of results over time, providing more credible conclusions regarding the associations examined. Moreover, the consistency in the data collection procedures between the baseline and follow-up assessments, which were supervised by the same trained staff, minimizes the potential for random measurement error.

## 5. Conclusions

This study demonstrated that increased MVPA durations and reduced screen time are correlated with improved diet quality and CRF. Substituting MVPA with an equivalent duration of screen time is positively linked with both diet quality and CRF. Moreover, diet quality mediates the relationship between MVPA, screen time, and CRF. Additionally, when MVPA is replaced with an equivalent duration of screen time, diet quality also serves as a mediator in the association with CRF. These findings underscore the significance of optimizing daily time allocation for MVPA and screen time, as well as enhancing diet quality to enhance physical fitness among school-aged children. Further research with extended follow-up periods is warranted to establish causal inferences regarding these associations.

## Figures and Tables

**Table 1:** Sample characteristics at baseline and at 9-month follow-up.

Variable	Baseline Sample (*n* = 1131)	Follow-Up Sample (*n* = 1268)
Sex, *n* (%)		
Boys	600 (53.1%)	638 (50.3%)
Girls	531 (46.9%)	630 (49.7%)
Mother’s education, *n* (%)		
Under high school	325 (28.7%)	356 (27.9%)
High school or above	806 (71.3%)	923 (72.1%)
Weight, kg	28.0 (24.8, 31.8)	29.9 (26.7, 34.5)
Height, cm	132.0 (128.3, 136.0)	136.5 (132.5, 140.4)
BMI, kg/m^2^	15.8 (14.7, 17.7)	15.9 (14.8, 18.0)
Age, years	8.5 (8.3, 8.8)	9.3 (9.0, 9.5)
Daily behavior time		
MVPA time, h/day	1.04 (0.46, 1.83)	1.31 (0.71, 2.18)
Sedentary time, h/day	2.83 (2.00, 3.71)	3.07 (2.19, 3.93)
Screen time, h/day	0.43 (0.17, 0.86)	0.48 (0.21, 0.79)
Sleep time, h/day	9.93 (9.47, 10.33)	10.04 (9.57, 10.48)
GDR score	11.0 (9.0, 13.0)	11.0 (9.0, 13.0)
FGDS	6.0 (4.0, 7.0)	6.0 (5.0, 7.0)
CRF, mL/kg/min	47.5 (46.0, 50.8)	48.3 (46.0, 51.8)

For continuous variables, if the Shapiro–Wilk test indicates non-normal distribution (*p* < 0.05), they are described using the median and interquartile range. If the variables follow a normal distribution, they are described using the mean and standard deviations. Abbreviations: BMI = body mass index; CRF = cardiorespiratory fitness; FGDS = Food Group Diversity Score; GDR = Global Dietary Recommendations; MVPA = moderate-to-vigorous physical activity.

**Table 2:** Associations of daily behavior activity with GDR score, FGDS, and CRF among school-aged children.

Outcomes	Daily Behavior Time	Model 1		Model 2		Model 3	
		β (95% CI)	*p*	β (95% CI)	*p*	β (95% CI)	*p*
GDR score	Baseline						
	MVPA time	0.11 (−0.01~0.23)	0.080	0.13 (0.01~0.25)	0.041	0.13 (0.01~0.25)	0.037
	Screen time	−0.09 (−0.22~0.05)	0.220	−0.06 (−0.20~0.08)	0.363	−0.09 (−0.24~0.06)	0.257
	Sedentary time	−0.001 (−0.06~0.06)	0.966	0.002 (−0.06~0.06)	0.938	0.02 (−0.05~0.08)	0.675
	Sleep time	−0.04 (−0.20~0.13)	0.668	−0.04 (−0.20~0.12)	0.656	−0.04 (−0.20~0.12)	0.599
	Follow-up						
	MVPA time	0.15 (0.05~0.23)	0.002	0.18 (0.09~0.26)	<0.001	0.18 (0.09~0.27)	<0.001
	Screen time	−0.41 (−0.60~−0.22)	<0.001	−0.32 (−0.51~−0.12)	0.002	−0.35 (−0.55~−0.15)	0.001
	Sedentary time	0.00 (−0.07~0.08)	0.908	0.01 (−0.07~0.08)	0.945	0.04 (−0.03~0.12)	0.268
	Sleep time	−0.04 (−0.18~0.10)	0.603	−0.07 (−0.21~0.07)	0.311	−0.07 (−0.21~0.07)	0.343
FGDS	Baseline						
	MVPA time	0.15 (0.06~0.25)	0.002	0.18 (0.08~0.27)	<0.001	0.18 (0.08~0.28)	<0.001
	Screen time	−0.07 (−0.18~0.04)	0.196	−0.01 (−0.12~0.10)	0.803	−0.004 (−0.13~0.12)	0.946
	Sedentary time	−0.03 (−0.08~0.02)	0.191	−0.02 (−0.07~0.03)	0.438	−0.02 (−0.08~0.03)	0.383
	Sleep time	−0.02 (−0.15~0.11)	0.782	0.01 (−0.12~0.14)	0.882	0.01 (−0.11~0.14)	0.856
	Follow-up						
	MVPA time	0.16 (0.09~0.23)	<0.001	0.18 (0.12~0.25)	<0.001	0.18 (0.12~0.25)	<0.001
	Screen time	−0.33 (−0.48~−0.18)	<0.001	−0.19 (−0.35~−0.04)	0.017	−0.21 (−0.36~−0.05)	0.013
	Sedentary time	−0.02 (−0.08~0.03)	0.421	−0.01 (−0.06~0.05)	0.788	0.01 (−0.04~0.07)	0.637
	Sleep time	0.01 (−0.10~0.11)	0.927	−0.05 (−0.16~0.06)	0.359	−0.05 (−0.15~0.06)	0.384
CRF	Baseline						
	MVPA time	0.40 (0.23~0.58)	<0.001	0.45 (0.28~0.61)	<0.001	0.45 (0.28~0.62)	<0.001
	Screen time	−0.24 (−0.43~−0.04)	0.018	−0.14 (−0.33~0.05)	0.157	−0.30 (−0.50~−0.09)	0.006
	Sedentary time	0.09 (0.00~0.18)	0.047	0.09 (0.00~0.18)	0.040	0.14 (0.04~0.23)	0.006
	Sleep time	−0.09 (−0.32~0.14)	0.424	−0.03 (−0.25~0.19)	0.784	−0.04 (−0.25~0.18)	0.745
	Follow-up						
	MVPA time	0.21 (0.08~0.34)	0.002	0.24 (0.12~0.36)	<0.001	0.24 (0.12~0.36)	<0.001
	Screen time	−0.41 (−0.69~−0.12)	0.005	−0.20 (−0.48~0.08)	0.153	−0.29 (−0.57~0.00)	0.050
	Sedentary time	0.05 (−0.06~0.16)	0.357	0.08 (−0.02~0.18)	0.129	0.11 (0.01~0.22)	0.042
	Sleep time	0.18 (−0.03~0.38)	0.097	0.12 (−0.08~0.31)	0.241	0.12 (−0.07~0.32)	0.217

Model 1 adjusted for none; Model 2 adjusted for sex, age, mother’s education, BMI, and schools, for follow-up data, further adjusted intervention groups; Model 3 adjusted for sex, age, mother’s education, BMI, and schools, additionally adjusted for the other three variables of daily behavior status. Abbreviations: BMI = body mass index; CI = confidence interval; CRF = cardiorespiratory fitness; FGDS = Food Group Diversity Score; GDR = Global Dietary Recommendations; MVPA = moderate-to-vigorous physical activity.

**Table 3:** Isotemporal substitution of daily movement behaviors in GDR score and FGDS.

Add 30 min/day	Remove 30 min/day	Baseline		Follow-Up	
		β (95% CI)	*p*	β (95% CI)	*p*
GDR score					
MVPA time	Screen time	0.11 (0.02~0.21)	0.024	0.26 (0.15~0.38)	<0.001
Sedentary time	Screen time	0.06 (−0.04~0.15)	0.227	0.20 (0.08~0.31)	0.001
Sleep time	Screen time	0.03 (−0.08~0.14)	0.631	0.14 (0.02~0.26)	0.026
MVPA time	Sedentary time	0.06 (−0.01~0.13)	0.117	0.07 (0.01~0.12)	0.020
FGDS					
MVPA time	Screen time	0.11 (0.03~0.19)	0.006	0.19 (0.11~0.28)	<0.001
Sedentary time	Screen time	0.01 (−0.06~0.09)	0.756	0.11 (0.02~0.20)	0.017
Sleep time	Screen time	0.02 (−0.06~0.11)	0.626	0.08 (−0.01~0.17)	0.099
MVPA time	Sedentary time	0.10 (0.05~0.16)	<0.001	0.08 (0.04~0.13)	<0.001
CRF					
MVPA time	Screen time	0.40 (0.26~0.53)	<0.001	0.26 (0.11~0.42)	0.001
Sedentary time	Screen time	0.24 (0.12~0.37)	<0.001	0.20 (0.03~0.36)	0.019
Sleep time	Screen time	0.15 (0.00~0.30)	0.052	0.20 (0.03~0.38)	0.021
MVPA time	Sedentary time	0.16 (0.06~0.25)	0.002	0.07 (−0.02~0.15)	0.114

Adjusted for sex, age, mother’s education, BMI, schools and intervention groups, additionally adjusted for the other three variables of daily behavior status. Abbreviations: BMI = body mass index; CI = confidence interval; CRF = cardiorespiratory fitness; FGDS = Food Group Diversity Score; GDR = Global Dietary Recommendations; MVPA = moderate-to-vigorous physical activity.

## Data Availability

The original contributions presented in the study are included in the article/supplementary material, further inquiries can be directed to the corresponding authors.

## Supplementary Materials

The following supporting information can be downloaded at: https://www.mdpi.com/article/10.3390/nu16162788/s1, Figure S1: Mediation analysis using baseline data. Contribution of screen time and MVPA on CRF through GDR score and FGDS, adjusting for potential confounders (age, sex, BMI, mother’s education, and school groups); Figure S2: Mediation analysis of GDR score and FGDS on the relationships between 30 min of daily screen time displacing MVPA time and CRF using baseline data, adjusting for potential confounders (age, sex, BMI, mother’s education, and school groups).
