# Supplementary material for: Isotemporal Substitution Effects of Daily Time Use on Cardiorespiratory Fitness of Children in the OptiChild Study: A Mediation Analysis with Diet Quality
Source: Nutrients. 2024 Aug 21;16(16):2788. doi: 10.3390/nu16162788 (PMC11357184; doi:10.3390/nu16162788)
Supplement: Supplementary file 1 [file nutrients-16-02788-s001.zip › nutrients-3100582-supplementary.pdf]

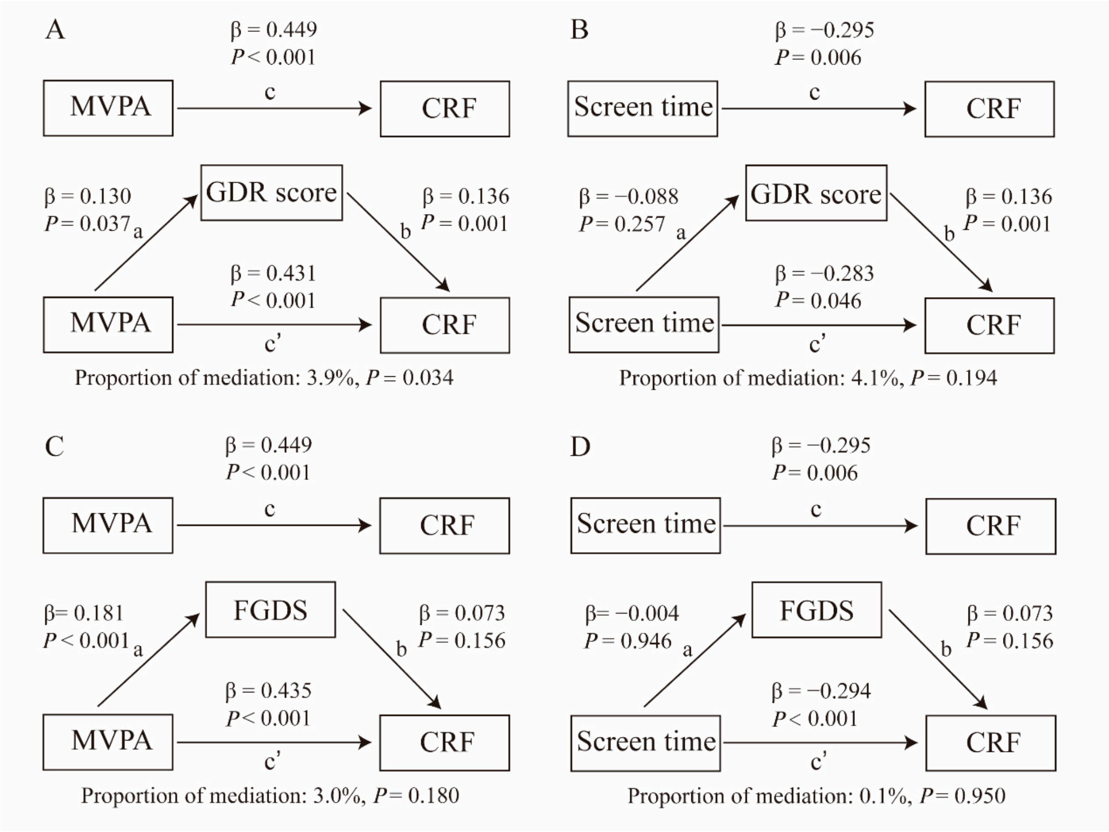

Figure S1. Mediation analysis using baseline data. Contribution of screen time and MVPA on CRF through GDR score and FGDS, adjusting for potential confounders (age, sex, BMI, mother's education, and school groups). (A) mediation role of GDR score between MVPA and CRF; (B) mediation role of GDR score be-tween screen time and CRF; (C) mediation role of FGDS between MVPA and CRF; (D) mediation role of FGDS between screen time and CRF.

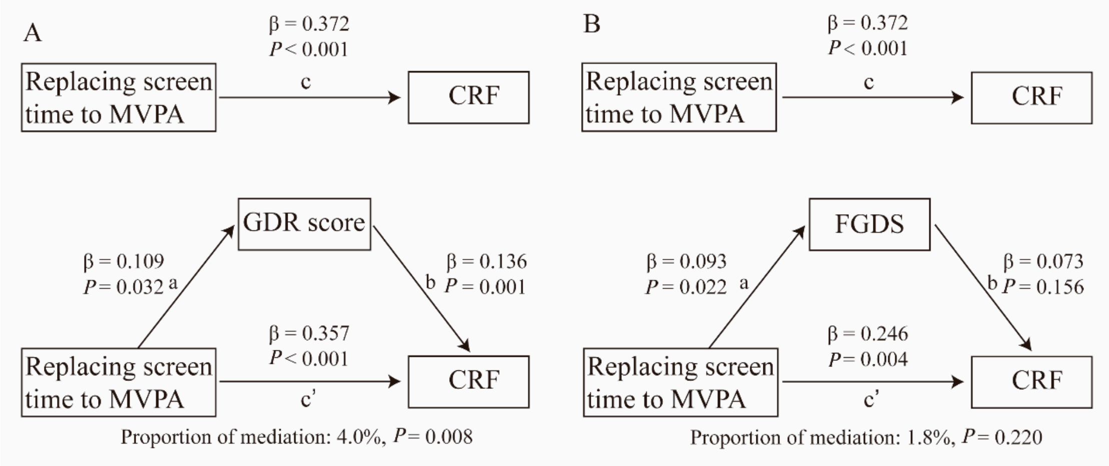

Figure S2. Mediation analysis of GDR score and FGDS on the relationships between 30 minutes of daily screen time displacing MVPA time and CRF using baseline data, adjusting for potential confounders (age, sex, BMI, mother's education, and school groups). (A) mediation analysis of GDR score; (B) mediation analysis of FGDS.
